# The Seasonal Metabolic Activity Cycle of Antarctic Krill (*Euphausia superba*): Evidence for a Role of Photoperiod in the Regulation of Endogenous Rhythmicity

**DOI:** 10.3389/fphys.2018.01715

**Published:** 2018-12-20

**Authors:** Fabio Piccolin, Lavinia Suberg, Robert King, So Kawaguchi, Bettina Meyer, Mathias Teschke

**Affiliations:** ^1^Section Polar Biological Oceanography, Alfred Wegener Institute, Helmholtz Centre for Polar and Marine Research, Bremerhaven, Germany; ^2^Institute for Chemistry and Biology of the Marine Environment, Carl von Ossietzky University of Oldenburg, Oldenburg, Germany; ^3^Department of the Environment and Energy, Australian Antarctic Division, Kingston, TAS, Australia; ^4^Antarctic Climate and Ecosystems Cooperative Research Centre, Battery Point, TAS, Australia; ^5^Helmholtz Institute for Functional Marine Biodiversity (HIFMB), University of Oldenburg, Oldenburg, Germany

**Keywords:** growth, oxygen consumption, enzyme activity, gene expression, clock genes, circannual clock

## Abstract

Antarctic krill (*Euphausia superba*), a key species in the Southern Ocean, reduce their metabolism as an energy saving mechanism in response to the harsh environmental conditions during the Antarctic winter. Although the adaptive significance of this seasonal metabolic shift seems obvious, the driving factors are still unclear. In particular, it is debated whether the seasonal metabolic cycle is driven by changes in food availability, or if an endogenous timing system entrained by photoperiod might be involved. In this study, we used different long-term photoperiodic simulations to examine the influence of light regime and endogenous rhythmicity on the regulation of krill seasonal metabolic cycle. Krill showed a seasonal cycle of growth characterized by null-to-negative growth rates during autumn-winter and positive growth rates during spring-summer, which was manifested also in constant darkness, indicating strong endogenous regulation. Similar endogenous cycles were observed for the activity of the key-metabolic enzyme malate dehydrogenase (MDH) and for the expression levels of a selection of metabolic-related genes, with higher values in spring-summer and lower values in autumn-winter. On the other side, a seasonal cycle of oxygen consumption was observed only when krill were exposed to simulated seasonal changes in photoperiod, indicating that light-related cues might play a major role in the regulation of krill oxygen consumption. The influence of light-regime on oxygen consumption was minimal during winter, when light-phase duration was below 8 h, and it was maximal during summer, when light-phase duration was above 16 h. Significant upregulation of the krill clock genes *clk*, *cry*2, and *tim*1, as well as of the circadian-related opsins *rh*1a and *rrh*, was observed after light-phase duration had started to decrease in early autumn, suggesting the presence of a signaling cascade linking specific seasonal changes in the Antarctic light regime with clock gene activity and the regulation of krill metabolic dormancy over the winter.

## Introduction

Antarctic krill *(Euphausia superba*, hereafter krill), a shrimp-like crustacean species, plays a central role in the Southern Ocean ecosystem, being both a major grazer of marine phytoplankton and a critical food item for whales, seals, birds, and fish, thereby linking primary production to higher trophic levels. Its circumpolar distribution shows a latitudinal range from 51°S to 70°S with more than 50% of Southern Ocean krill stocks located in the southwest Atlantic sector and the region of the West Antarctic Peninsula (Atkinson et al., 2004). These areas are currently experiencing some of the most rapid anthropogenic-driven warming on Earth, resulting in a 1°C increase of the surface summer temperature of the adjacent ocean since 1950 (Meredith and King, 2005). There have been indications that the distribution and abundance of krill in the southwest Atlantic sector have already been altered significantly as a result of changes in primary productivity associated with the decline in sea ice (Atkinson et al., 2004). In addition, krill are the object of a developing fishery and concern has been expressed about the future sustainability of Antarctic krill fisheries in a changing environment (Schiermeier, 2010). The central position of krill in the food web, the ongoing environmental changes in its habitat, and increasing commercial interest emphasize the urgency to understand the adaptability of krill to its environment.

The Southern Ocean pelagic environment is characterized by extreme seasonal changes in environmental factors such as day length (photoperiod), light intensity, sea ice extent, and food availability. Almost complete darkness in winter, when most of the Southern Ocean is covered by sea ice, alternates with near constant daylight in summer. This, in turn, means almost no food in the water column during winter with less than 0.1 μg L^-1^ Chl *a* (chlorophyll-*a*) and primary production lower than 1 mg C m^-2^ d^-1^, in contrast to massive phytoplankton blooms of >1000 mg C m^-2^ d^-1^ in spring and early summer resulting in available Chl *a* concentrations of more than 10 μg L^-1^ (Atkinson et al., 2002; Vernet et al., 2012). Consequently, biological timing that ensures regulation of krill’s physiology and behavior in reaction to annual fluctuations of biologically significant factors is particularly advantageous and likely to be a major feature determining the success of krill in the Southern Ocean. Many high latitude species have developed circannual (approximately a year) oscillations that enable them to anticipate and prepare for forthcoming environmental changes and synchronize seasonal events (e.g., reproduction) to environmental fluctuations (Jørgensen and Johnsen, 2014).

Indeed, krill has evolved a reproductive seasonal cycle with a peak of full sexual maturity and reproduction in the favorable summer months (December–January) and a trough (regression of sexual maturity) during winter months (May–June) (Kawaguchi et al., 2007). In the laboratory, this cycle can be maintained independently without direct control of factors such as food, light or temperature (Thomas and Ikeda, 1987; Kawaguchi et al., 2007; Brown et al., 2013), indicating that krill passes through a fundamental inherent seasonal transition in maturity development. Other experiments showed that light conditions of prolonged photoperiod can force krill into maturity while shortened photoperiods force animals into regression of maturity faster than under a natural light-dark regime (Teschke et al., 2008; Brown et al., 2011). These experiments strongly indicate that the overt cycle of maturity represents an endogenous annual rhythm (circannual), controlled by an endogenous timing system in krill (endogenous clock) in which photoperiod acts as a main Zeitgeber (entraining cue), synchronizing the clock with the natural year.

Similarly, adult krill show cycles of growth and metabolic regulation throughout the year, with highest growth rates, feeding and metabolic activity during the summer months (December–January) and quiescent-like reduced growth, feeding and metabolic activity during winter (May–June). This process is thought to represent a major overwintering mechanism during times when food availability is low (Quetin and Ross, 1991; Cullen et al., 2003; Meyer, 2012), however, the underlying mechanisms leading to this seasonal rhythm are still not fully understood.

**FIGURE 1:**
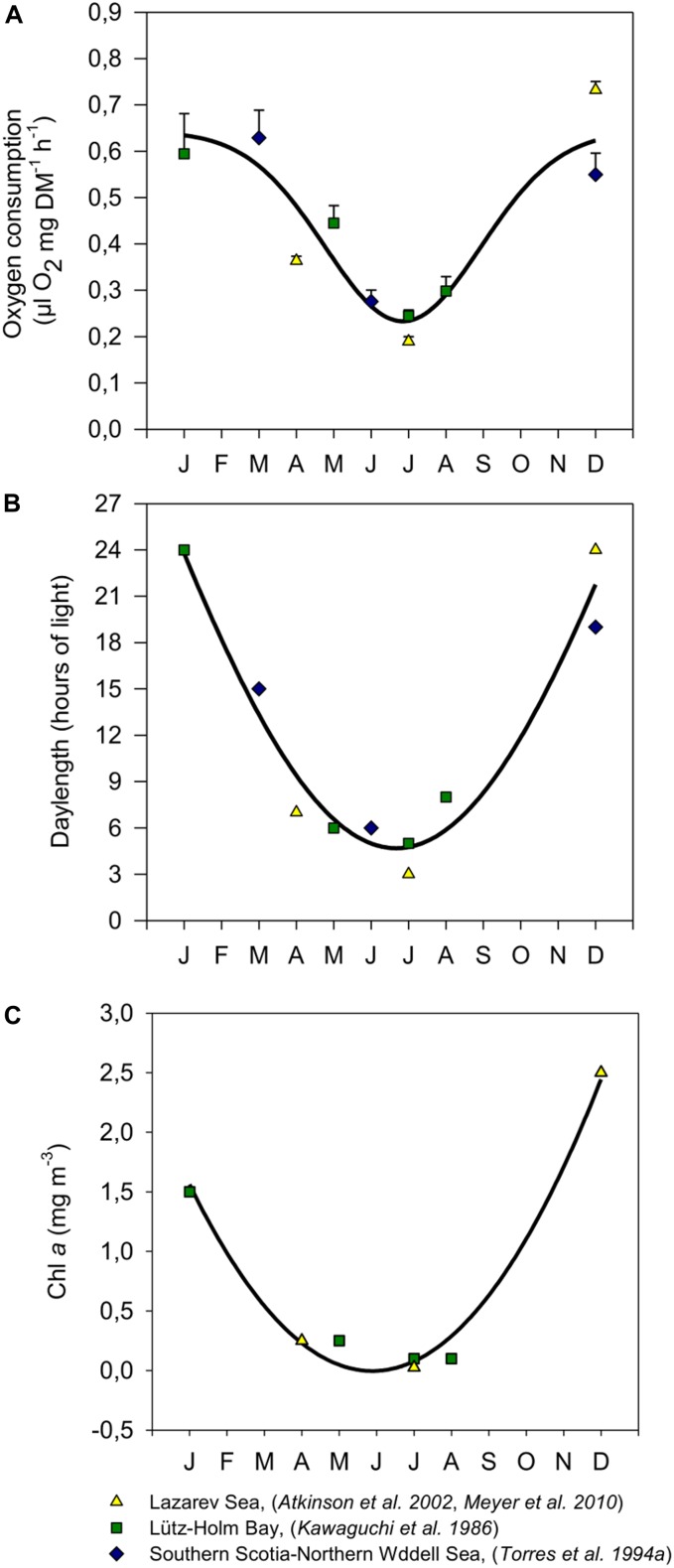
Seasonal respiration rates of adult krill from different study sites in the Southern Ocean **(A)**, and the corresponding photoperiods **(B)** and Chl *a* concentrations **(C)** at those sites during measurements, modified after Meyer et al. (2010). Values in **A** were expressed as means ± SEM (January: *n* = 3, March: *n* = 2, April: *n* = 16, May: *n* = 5, June: *n* = 3, July: Atkinson et al. (2002), Meyer et al. (2010): *n* = 17; Kawaguchi et al. (1986): *n* = 5, August: *n* = 3, December: Atkinson et al. (2002), Meyer et al. (2010): *n* = 8; Torres et al. (1994): *n* = 3). For respiration rates **(A)** photoperiods **(B)** and Chl *a* concentrations **(C)** a non-linear regression curve was fitted (**A**: *R* = 0.926; *P* =? 0.0001; *n* = 65, **B**: *R* = 0.974; *P* = 0.0003; *n* = 10, **C**: *R* = 0.992; *P* = 0.0034; *n* = 7). Note that no Chl*a* values were available for Torres et al. (1994).

A central question is whether such metabolic shifts observed in krill over the seasons simply reflect a change in ingestion rate (i.e., food availability), or whether these are the result of an endogenous adaptive seasonal cycle driven by photoperiod, similar to what has been shown for the maturity cycle. A compilation of data from different investigations on the seasonal metabolic activity of krill in different regions of the Southern Ocean (Figure 1A) and the corresponding cycles of photoperiod and Chl *a* concentration (Figures 1B,C) demonstrate the correlation of these parameters and imply that both, the seasonal cycle of photoperiod, and food availability in the environment have the potential to play a prominent role as modulating factor for seasonal changes in metabolic rates of krill.

Long-term experiments testing the effect of photoperiod on krill in the laboratory demonstrated that feeding and metabolic activity were affected by different simulated light-dark cycles (LD) irrespective of food supply, suggesting that simulations of prolonged photoperiod can force animals into a state of increased metabolic activity and vice-versa (Teschke et al., 2007). Moreover, molecular analyses (Seear et al., 2009) revealed differential gene expression of target genes (i.e., those involved in metabolism) in response to photoperiodic changes, indicating a signaling cascade that link the photoperiod cue to the target response. However, despite these studies, up to date experimental evidence for the causative connection between the cycle of physiological and metabolic activity of krill throughout the year and the seasonal course of photoperiod is still elusive.

The primary aims of this study were (1) to examine whether and to what extent different physiological life cycle-parameters of krill are synchronized to the seasonal cycle of photoperiod, irrespectively, of food supply; and (2) to investigate the involvement of an endogenous circannual rhythm in the regulation of the seasonal metabolic cycle in krill. In addition to these, a secondary aim of the study was (3) to further characterize the relationship between the endogenous rhythm and the seasonal photoperiodic cycle, by investigating the metabolic response of krill to a seasonal shift in the photoperiodic cycle. To implement these aims, we exposed krill to long-term simulations of different photoperiodic cycles and we monitored seasonal changes in a set of metabolic-related parameters including growth, oxygen consumption, enzyme activity, and gene expression.

## Materials and Methods

### Ethics Statement

All animal work has been conducted according to relevant national and international guidelines. Krill catches, welfare and experimentation were based on permission of the Department of Environment and Heritage (DEH) of the Australian Government and were conducted in accordance with the Antarctic Marine Living Resources Conservation Act 1981 (AMLR, permit number: 06_09_2220) and the Environment Protection And Biodiversity Conservation Act 1999 (EPB, permit number: WT2007-1480).

### Collection of Animals in the Field and Maintenance in the Laboratory

*Euphausia superba* were collected during the voyage V1 07/08 of RSV *Aurora Australis* by means of several Rectangular Midwater Trawls (pelagic net, RMT 8) in the upper 200 m of the water column between 65° 19′S, 125° 37′E (September 17, 2007) and 64° 08′S, 119° 16′E (October 9, 2007). After collection, krill were maintained on board in 200 L tanks on board with continuous supply of seawater in 0°C laboratory, with dim light and virtually no food, other than the small amount contained within the ambient water flow. After arrival in Hobart, Tasmania, krill were transferred to the Australian Antarctic Division (AAD) marine research aquarium and kept in a 1670 L holding tank connected to a 8000 L chilled seawater recirculation system. In the water system of the aquarium, water temperature was maintained at 0.5°C, salinity was kept at around 34.5 PSU, pH was kept at around 8.0, ammonia levels were generally below the detection limit (<0.015 mg/L NH_3_-N), nitrate levels were usually around 1.78 mg/L NO_3_-N, and nitrite levels were always below detection limit (<0.6 mg/L NO_2_-N). Light was provided by fluorescent tubes and controlled by a computer-regulated timer system which provided a simulation of the natural photoperiodic cycle occurring at 66°S. For a more detailed description of the aquarium see King et al. (2003) and Kawaguchi et al. (2010).

### Experimental Setup

#### Experimental Design

To investigate whether and to what extent different physiological life cycle-parameters of krill are synchronized to the seasonal cycle of photoperiod, irrespective of food supply (aim n°1), we exposed krill to a long-term simulation of the natural annual course of Antarctic photoperiod corresponding to 66°S (hereafter denoted as LD treatment). In LD, for 1 year krill were exposed to monthly changes in photoperiod (Table 1), mimicking the natural photoperiodic conditions experienced by krill in the corresponding month in the Southern Ocean, at the reference latitude of 66° South (66°S). 66°S was chosen because of the significant seasonal changes in metabolic activity usually displayed by wild krill at this latitude (Meyer, 2012). To avoid seasonal signals coming from changes in food conditions, food availability was kept constant throughout the treatment.

**Table 1:** Daily duration of the light phase (hours) in the different experimental months and different experimental treatments.

month	LD	LD 1/2	DD
00_December	24	24	0
01_January	21	16	0
02_February	16	9	0
03_March	13	3	0
04_April	9	8	0
05_May	6	15	0
06_June	3	24	0
07_July	4	–	0
08_August	8	–	0
09_September	11	–	0
10_October	15	–	0
11_November	19	–	0
12_December	24	–	0


To investigate the involvement of an endogenous circannual rhythm in the regulation of the seasonal metabolic activity cycle in krill (aim n°2), we exposed krill to a long-term constant darkness treatment (hereafter denoted as DD treatment). In DD, for 1 year krill were exposed to constant darkness (Table 1), and food availability was kept at a constant high level throughout the treatment as in LD. The DD treatment represents the “free-running” condition, where all potential external seasonal changes (temperature, food, and light) are avoided and the presence of an underlying endogenous rhythm can be revealed.

To investigate the metabolic response of krill to a shift in the seasonal photoperiodic cycle (aim n°3), we exposed krill to a long-term simulation of the annual course of Antarctic photoperiod at 66°S, but shortened into a 6 months period (hereafter denoted as LD1/2 treatment) (Table 1). The LD1/2 treatment was added to further characterize the relationship between the endogenous rhythm and the seasonal photoperiodic cycle: if photoperiod acts as strong *Zeitgeber* (i.e., entraining cue), we would expect a corresponding shift in the metabolic cycle, whereas if the endogenous component prevails, the metabolic cycle should remain mostly unaffected.

To monitor seasonal changes in krill metabolic activity in the LD and DD treatments we used a multi-level approach, measuring temporal changes in total body length, oxygen consumption, levels of activity of the key metabolic enzyme Malate Dehydrogenase (MDH) and levels of expression of a selection of key metabolic genes. This was done because for LD and DD we wanted to provide a detailed description of the metabolic response of krill under the different light regimes. Conversely, in the LD1/2 treatment we only measured temporal changes in oxygen consumption, because we were interested in a quick analysis of the overall metabolic response of krill to a shift in the photoperiodic cycle.

#### Experimental Tanks

The experiment started in December 2009 by separating approximately 900 adult krill of mixed sex from the holding tank into three cylindrical 100 L tanks (300 krill each), filled with 0.5°C seawater. The seawater supply was connected to the re-circulating sea-water facility of the aquarium to guarantee identical water quality and temperature for all experimental stock throughout the study (see section “Collection of Animals in the Field and Maintenance in the Laboratory”). Each tank was surrounded by a black lightproof plastic container with a sliding door at the front to create a separate light compartment. Light inside the containers was provided by fluorescent tubes (Osram L18W/640 Cool White) covered with a gel filter (ARRI, Marine Blue 131) simulating light attenuation at 30 m depth. Photoperiod and light intensity were controlled through separate channels by the same PC-controlled timer system used for the rest of the aquarium (see section “Collection of Animals in the Field and Maintenance in the Laboratory”).

#### Feeding

All experimental tanks were exposed to the same feeding regime as used for the rest of the aquarium population. Krill were fed daily with a mixture of living algae at the final concentration of 1.5 × 10^4^ cells ml^-1^ of the pennate diatom *Phaeodactylum tricornutum*, 2.2 × 10^4^ cells ml^-1^ of the chlorophyte *Pyramimonas gelidicola* and 2 × 10^4^ cells ml^-1^ of the cryptophyte *Geminigera cryophila*. Instant algae were added to yield final concentrations of 1 × 10^4^ cells ml^-1^ of *Thalassiosira weissfloggii* (1200TM, CCMP1051/TWsp., Reed Mariculture, United States), 5.1 × 10^4^ cells ml^-1^ *Isochrysis sp.* (1800TM, Reed Mariculture, United States), and 4.8 × 10^4^ cells ml^-1^ *Pavlova sp.* (1800TM, Reed Mariculture, United States). Krill also received 1 g per tank per day of nutritional supplements (0.5 g of Frippak #1 CAR, 0.5 g of Frippak #2 CAR, INVE, Thailand). After the phytoplankton mix was added, water flow into the tank was shut off for 2 h to enable krill to feed on the food mixture. This feeding regime has been used successfully in several experimental trials at the AAD prior to this study and it has been found to guarantee good condition of krill in long-term laboratory experiments with low mortality and high feeding rates (King et al., 2003; Kawaguchi et al., 2010). Feeding during dark phases was conducted under dim red light. Feeding was suspended on the day of each sampling until sampling was complete.

#### Light Treatments

Following the experimental design introduced in the section “Experimental Design,” the three tanks were exposed to one of the following long-term photoperiodic treatments, respectively: (1) LD treatment, simulation of the natural annual course of Antarctic photoperiod corresponding to 66°S; (2) DD (dark/dark) treatment, constant darkness; and (3) LD1/2 treatment, simulation of the annual course of Antarctic photoperiod corresponding to 66°S, but shortened into a 6 months period (Table 1). In the LD and LD1/2 treatments, a midday maximum of 100 lux at the surface of the tanks was set during light phases. The transition between light and dark phases happened gradually to simulate sunrise and sunset. Both photoperiodic simulations started at a light/dark ratio in agreement with that of the holding tank from which they originated, ensuring optimal acclimation transition into the experimental treatments. Photoperiod in LD was then adjusted at the beginning of each month while photoperiod in LD1/2 was changed every 2 weeks.

### Oxygen Consumption and Body Length Measurements

To examine changes over time in the overall metabolic activity of krill under the different light regimes (LD, DD, and LD1/2), oxygen consumption of three individual krill was determined every month in each tank. Krill were incubated individually for 24 h in 2 L bottles, which were completely filled with filtered seawater (0.1 μm pore size), sealed with parafilm and placed back into the corresponding experimental tanks to maintain the experimental temperature at 0.5°C. One bottle of the same volume without krill was used as control for each tank. At the end of the incubation time, three subsamples were siphoned out from each incubation bottle and transferred into separate 50 ml Winkler bottles using a glass tube, according to Atkinson et al. (2002). Oxygen concentration was measured after immediate fixation for Winkler titrations as described in Meyer et al. (2002), using a 702 SM Titrino (Metrohm). The decrease in oxygen concentration for all experiments was <10%, which did not affect krill behavior and hence respiration rates (Johnson et al., 1984). Oxygen consumption was calculated in μl O_2_^∗^mg^-1^ DM ^∗^ h^-1^ using the formula

$$R=(O_{c}-O_{k})V/\left(m_{k}\times t\right)$$

as described in Atkinson et al. (2002), where *R* is the oxygen consumption (also called “respiration rate”), *O*_c_ is the O_2_ concentration in the control bottle at the end of the experiment (μl O_2_ L^-1^), *O*_k_ is the corresponding value in the experimental bottle, *V* is the volume before subsampling (L), *m*_k_ is the individual dry mass (DM) of the experimental krill (mg), and *t* is the experimental duration (h).

In order to avoid additional stress for the experimental animals, which for LD and DD were planned to use for further analyses, the individual dry mass (DM) in mg was calculated from the total length of the animals as described in Brown et al. (2013). Total body length of each krill was measured from the anterior tip of the rostrum to the posterior end of the uropods, excluding their terminal setae. For the LD and DD treatments, the animals were then individually snap-frozen in liquid nitrogen and stored at -80°C for later enzyme activity and gene expression measurements (see below).

### Malate Dehydrogenase (MDH) Activity Measurements

Malate dehydrogenase is a key metabolic enzyme which plays a major role in the citric acid cycle as well as in other aspects of overall metabolism and has been used previously as a proxy for overall krill metabolic rate (Donnelly et al., 2004; Teschke et al., 2007). To analyze MDH activity (MDH; EC 1.1.1.37), the fifth abdominal segment of the frozen animals was used. The dissection and the determination of the fresh weight (fw) took place on a chilled stage to avoid thawing. The tissue was homogenized in pre-weighed 2 mL tubes containing ceramic beads of 1.4 and 2.8 mm diameter (Precellys^®^) in ice-cold deionized water at a concentration of 100 mg fw ml^-1^, which corresponds to a dilution of 1:10. Homogenization was performed using the Precellys^®^ 24 homogenizer with two agitation intervals of 15 s at 5000 rpm and one pause of 10 s between intervals. A constant temperature of 4°C within the homogenization chamber was maintained using a Precellys^®^ cooling module pre-filled with liquid nitrogen. The homogenates were centrifuged for 15 min at 14000 rpm (4°C) and the supernatants were then transferred into new reaction tubes and stored at -80°C until analysis. MDH activity was determined according to Teschke et al. (2007) in a 96-well plate in technical triplicates. To 180 μl reaction buffer (0.1 M KH_2_PO_4_, pH 7), 6.7 μl NADH (7 mM), and 6.7 μl homogenate (diluted 1:30 in reaction buffer) were added. The reaction was started with 6.7 μl Oxalacetate (12 mM) and monitored at 25°C, 340 nm for 5 min. The activity was expressed as U g fw^-1^ ( = μmol min^-1^ g fw^-1^) using the extinction coefficient ε340 = 6.22 L mmol^-1^ cm^-1^.

### Gene Expression Measurements

#### Sampling

For the gene expression measurements krill were sampled from the LD and DD treatments in December (2009), February, June, and August (2010). Those moments of the years were selected because they represent relevant phases during the seasonal metabolic cycle of krill. December represents the mid-summer phase, when krill feeding and metabolic activity are generally upregulated; February represents the early-autumn phase, when metabolic activity usually starts to decrease toward winter; June represents the mid-winter phase, when krill are usually found in a state of metabolic quiescence; and August represents the late-winter phase, when krill usually start to emerge from quiescence and increase their metabolism back toward spring and summer levels. The sampling took place always at the beginning of the 3rd week of the corresponding month, and in order to average out the effect of daily variability, six animals were collected every 3 h over the 24 h cycle (6AM, 9AM, 12PM, 3PM, 6PM, 9PM, 12AM, and 3AM). During sampling, feeding was suspended and during dark phases sampling was conducted under dim red light. The sampled animals were immediately snap-frozen in liquid nitrogen and stored at -80°C for molecular analyses.

#### RNA Extraction and cDNA Synthesis

Total RNA was extracted from krill heads to determine relative levels of expression of target genes. Frozen krill heads were cut off on dry ice and immediately transferred in Precellys^®^ tubes containing the TRIzol^®^ reagent. The tissue was homogenized at 4°C using a Precellys^®^24 tissue homogenizer (Bertin Instruments) connected to a Cryolys cooling element. The homogenate was removed from the Precellys^®^ tubes and treated with chloroform/isopropanol for phase separation and precipitation. The RNA pellet was washed two times with 75% EtOH and resuspended in RNase-free water. Total RNA was checked for concentration and purity using a NanoDrop^TM^2000 UV-Vis Spectrophotometer (ThermoScientific) and for integrity using an Agilent 2100 Bioanalyzer system (Agilent Technologies). To prevent genomic contamination, all samples were treated with the TURBO DNA-Free kit from Ambion (ThermoScientific). After DNA removal, for each sample 2 μg of total RNA were retro-transcribed to cDNA using the RevertAid H Minus Reverse Transcriptase kit from Invitrogen (ThermoScientific) to a final volume of 50 μl per sample (40 ng/μl).

#### Primer Design and qPCR

Gene expression was measured using custom-designed TaqMan^®^ Low-Density Array Cards (ThermoScientific). Primers for qPCR analysis were designed around sequences of interest using the Custom TaqMan^®^ Assay Design Tool (ThermoScientific). To examine the effect of the seasonal photoperiodic cycle on krill metabolic gene expression, we investigated seasonal changes in the expression levels of six genes involved in carbohydrate metabolism (*cs*, *pfk*6), lipid synthesis (*acc*), amino acid metabolism (*gldh*), protein synthesis (*ef*1a), and energy metabolism (*atp*) (Supplementary Table S1). Moreover, to investigate the involvement of the putative light-entrained timekeeping mechanism at the molecular level, we included in the analysis three genes related to the krill circadian clock (*clk*, *cry*2, *tim*1) and three genes related to krill light perception (*rh*1a, *rh*6, and *rrh*) (Supplementary Table S1). All sequences used for primer design are available online in the recently published krill database at http://krilldb.bio.unipd.it (Sales et al., 2017). For the LD treatment and the February DD sample, all collected krill were analyzed (six krill per eight time points, total *n* = 48), whereas for the December, June, and August DD samples, due to high analytical costs and budget limitation, only four krill at four time-points (6AM, 12PM, 6PM, and 12AM) were used, for a total *n* = 16.

For each biological replicate we mixed 20 μl of cDNA (800 ng), 30 μl of RNase-free water and 50 μl of TaqMan Gene Expression Master Mix (ThermoScientific), for a final volume of 100 μl which was loaded on the cards. The cards were analyzed using a ViiA^TM^ 7 Real-Time PCR System (ThermoScientific). Due to technical failures affecting one card run (ineffective sealing of the card prior to analysis), six additional biological replicates could not be included in the final analysis, giving us the following final sample sizes: December in DD, *n* = 16; December in LD, *n* = 47; February in DD, *n* = 48; February in LD, *n* = 48; June in DD, *n* = 16; June in LD, *n* = 43; August in DD, *n* = 16; August in LD, *n* = 48. Standard curves were conducted to calculate the efficiency of each primer pair on the card.

#### Normalization and Relative Quantification

The levels of transcription of the target genes were normalized and quantified using the modified 2^-ΔΔ*Ct*^ method proposed by Hellemans et al. (2007). In the classic 2^-ΔΔ*Ct*^ method, one reference gene, which is assumed to be expressed at a constant level across treatments, is used to normalize the expression values of the target genes in the different treatments. The modified 2^-ΔΔ*Ct*^ method represents an improvement, as it takes into account the gene-specific amplification efficiency of the primers used, and allows for the combination of multiple reference genes during the normalization (Hellemans et al., 2007). To select the most stable genes to use in the normalization procedure, we tested three candidate reference genes using Normfinder (Andersen et al., 2004). Our candidate reference genes were *ubiquitin carboxyl-terminal hydrolase* 46 (*usp*46), *ribosomal protein* S13 (*rps*13) and *ribosomal protein* L32 (*rpl*32) (Supplementary Table S1). *Usp*46 had showed constant mRNA levels in previous analyses of krill exposed to different LD/DD conditions (Biscontin et al., 2016), while *rps*13 and *rpl*32 had showed constant mRNA levels in previous experiments involving manipulation of photoperiod in the Lepidoptera *Plutella xylostella* (Fu et al., 2013). Following our stability analysis, *usp*46 was selected as the most stable reference gene candidate, showing a variability of ≤0.25 *C*q (quantification cycle) in the LD treatment and ≤0.98 *C*q in the DD treatment, and was therefore used as the reference in the normalization procedure. Normalized relative quantities (NRQs) were calculated by selecting as a baseline the sample showing the lowest *C*q-value among both treatments.

### Statistical Analyses

All data analyses were conducted using RStudio version 1.0.136 (RStudio Team, 2016). Following the experimental design described in 2.3.1, we conducted separate analyses for: (1) the temporal patterns of body length, MDH activity and oxygen consumption in LD and DD; (2) the temporal pattern of oxygen consumption in LD1/2; (3) the relationship between light-phase duration and oxygen consumption in LD and LD1/2; and (4) the seasonal changes in gene expression in LD and DD. In particular, the analysis of oxygen consumption in LD1/2 was kept separated due to (1) different data coverage in relation to the other two treatments (only three experimental months overlapped between LD1/2 and the other two treatments, and the compressed light cycle in LD1/2 only covered half of the total length of the time series for LD and DD); and (2) potentially different effects of the treatments (the LD and DD treatments tested the effect of different long-term light regimes on the seasonal metabolic cycle of krill, whereas the LD1/2 treatment tested the effect of a photoperiodic shift on the overall seasonal metabolic response of krill). To compare the effect of photoperiod on krill metabolic output between the LD and LD1/2 treatment, the relationship between hours of light and oxygen consumption (light:oxy) was modeled together (LD and LD1/2). This was possible since all light conditions were present in both treatments. Finally, in the LD and DD treatments the seasonal changes in gene expression were tested separately from the other parameters because of the different sampling rate (monthly vs. seasonal).

#### Comparison of Temporal Patterns of Body Length, MDH Activity and Oxygen Consumption in the LD and DD Treatments

To investigate differences in the temporal patterns of krill body length, MDH activity and oxygen consumption between LD and DD, a generalized additive model (GAM) with a Gaussian distribution was used. An additive model was chosen over a linear one to resolve the non-linear relationship of the response variables over time. The GAM took the structure as specified by Hastie and Tibshirani (1987) and was fitted using the *gam* function in the “mgcv” package (Wood, 2011). Prior to the modeling process, temporal autocorrelation was examined using the *acf* function in R. Time series are often subject to latitudinal dependencies between data points and not accounting for autocorrelation can result in biased estimates of model parameters (Peres-Neto, 2009). Although in some cases autocorrelation was found, it was resolved during the modeling process by the explanatory variables and was not evident in the residuals during model validation. Therefore, no temporal autocorrelation term was included in the final model.

Smoothed terms (continuous, non-linear variables) were fitted as regression splines (variable: experimental *month*). In order to avoid overfitting, the smoothing parameters were manually restricted to *k* ≤ 6 when necessary. Differences in temporal patterns between the two treatments (LD, DD) were implemented using the *by*-argument, which allows for the creation of separate smoother functions for each level of the *treatment* factor over the temporal variable *month*. Hence, separate parameter estimates for variable *month* for each *treatment* level were obtained. In order to test whether the temporal patterns between treatments were significantly different, the fit of the model with separate smoother functions for each *treatment* level was compared to one with a single smoother function for variable *month*, using the *anova* function and F-statistic. In addition, Akaike Information Criterion (AIC) (Akaike, 1981) was consulted to check whether a model with separate functions for each treatment level enhanced model fit. In the case of oxygen consumption, one extreme observation was excluded from the analysis, as it significantly improved the model fit. Model fit was examined by means of residual analysis.

#### Temporal Pattern of Oxygen Consumption in LD1/2

The temporal pattern of oxygen consumption in LD1/2 was modeled as described in the section “Comparison of Temporal Patterns of Body Length, MDH Activity and Oxygen Consumption in the LD and DD Treatments.” However, due to the skewed distribution of the response variable, a gamma distribution was used. Furthermore, the number of knots was manually set to *k* = 4. To compare oxygen consumption between LD and LD1/2 in those months where data from both treatments are available (i.e., January-March-June), we used the non-parametric Wilcoxon Rank Sum test implemented by the *wilcox.test* function in R.

#### Relationship Between Light-Phase Duration and Oxygen Consumption in LD and LD1/2

As the relationship between light and oxygen consumption for LD and LD1/2 was found to be non-linear, a GAM was implemented as described in the section “Comparison of Temporal Patterns of Body Length, MDH Activity and Oxygen Consumption in the LD and DD Treatments.” Again, a single extreme observation was removed as it significantly improved the model fit. In the model, the smoother function for *light* was included together with an interaction term for *treatment* (levels: LD and LD1/2), which considered the different relationships between oxygen consumption and light for each of the two treatments (LD and LD1/2). Differences between the treatments were investigated using AIC and the *anova* function on the model fits as described in the section “Comparison of Temporal Patterns of Body Length, MDH Activity and Oxygen Consumption in the LD and DD Treatments.”

#### Seasonal Changes in Gene Expression in LD and DD

To analyze changes in gene expression among seasonal samples in LD and DD, we used the non-parametric Kruskal-Wallis (K-W) Rank Sum test implemented by the *kruskal.test* function in R. The K-W test does not assume normality and works well with unequal sample sizes. To correct for multiple testing, we applied the false discovery rate (fdr) method implemented by the *p.adjust* function in R. The false discovery rate method is a less conservative correction method compared to the familywise error rate method implemented for example by the Bonferroni correction, meaning that it provides a greater statistical power at the cost of a less stringent control over Type I errors. To further characterize the seasonal expression patterns we did *post hoc* pairwise comparisons between seasonal samples (within treatment) using Dunn’s (1961) method implemented by the *kwAllPairsDunnTest* function in the “PMCMRplus” package (Pohlert, 2018), using the Holm (1979) method to correct the *p*-values. Finally, to examine in more detail the effect of light on gene expression in the different seasons, we used the non-parametric Wilcoxon Rank Sum test implemented by the *wilcox.test* function in R to test differences between LD and DD within the different months (December-February-June-August), using Holm’s method to correct the *p*-values.

## Results

### Temporal Patterns of Krill Body Length in LD and DD

**FIGURE 2:**
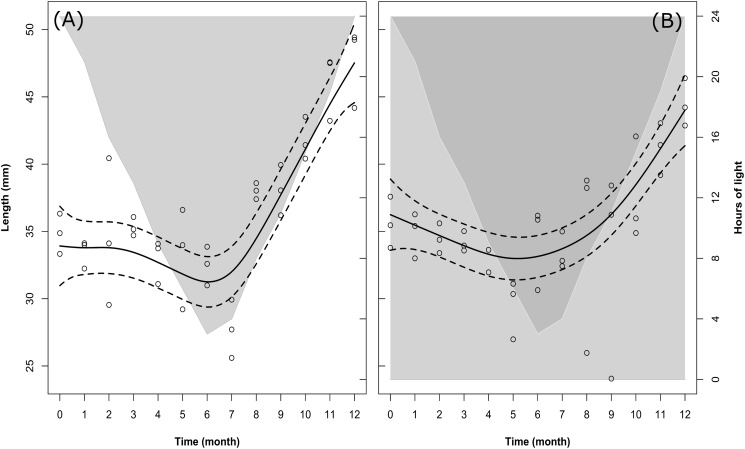
Changes in krill body length over time in the LD **(A)** and DD **(B)** treatments. Solid dark lines represent the model fit; dotted lines represent confidence intervals around the fit; circles represent individual measurements. In LD **(A)**, light-gray shaded area vs. white area represents temporal changes in the duration of dark phase vs. light phase. In DD **(B)**, dark-gray shaded area vs. light-gray shaded area represents temporal changes in the duration of dark phase vs. subjective light phase (i.e., the phase which was exposed to light during the corresponding month in the LD treatment).

In both treatments (LD and DD) there were significant changes in the body length of the sampled krill over time (Table 2, M1), but no significant differences were observed for the model fit between the two treatments (Model fit comparison with Anova: *F*-value = 2.4; *p-*value = 0.07). In fact, following the model fit (solid lines in Figures 2A,B), the temporal pattern was very similar between both treatments, with a slight decrease in krill body length occurring during the first half of the experiment, followed by an intense increase occurring during the second half (Figures 2A,B).

**Table 2:** GAM statistics for parametric coefficients (estimates, standard errors (SE), *F*-or *t*-values and *p*-values), explained variance (Deviance or *R*^2^) and non-parametric terms (smooth; estimated degrees of freedom (edf), *F*-statistic and *p*-values).

Intercept M1	Estimate	SE	*t*-value	*p-value*	Deviance
**Length**	36.04	0.4	100.4	≤*0.001*	68.50%
**Smooth**	**edf**		***F*-value**	***p-value***	
*Month LD*	4.04		20.1	≤*0.001*	
*Month DD*	2.82		12.33	≤*0.001*	
**Intercept M2**	**Estimate**	**SE**	***t*-value**	***p-value***	**Deviance**
**Oxygen**	0.3	0.018	16.79	≤*0.001*	43.10%
**Smooth**	**edf**		***F*-value**	***p-value***	
*Month LD*	3.69		5.01	*0.003*	
*Month DD*	1		0.69	*0.41*	
**Intercept M3**	**Estimate**	**SE**	***t*-value**	***p-value***	**Deviance**
**Oxygen**	0.88	0.005	179	≤*0.001*	62.60%
**Smooth**	**edf**		***F*-value**	***p-value***	
*Month LD 1/2*	2.08		5.17	*0.027*	
**Intercept M4**	**Estimate**	**SE**	***t*-value**	***p-value***	**Deviance**
**Oxygen**	0.3	0.015	20.56	≤*0.001*	69.60%
**Smooth**	**edf**		***F*-value**	***p-value***	
*Light LD*	5.21		10.93	≤*0.001*	
*Light LD1/2*	1.05		1.17	*0.3*	
**Intercept M5**	**Estimate**	**SE**	***t*-value**	***p-value***	**Deviance**
**MDH**	202.1	4.34	46.55	≤*0.001*	39.10%
**Smooth**	**edf**		***F*-value**	***p-value***	
*Month LD*	4.63		5.96	*0.0003*	
*Month DD*	3.43		2.5	*0.053*	


***M1**, body length over time (LD–DD); **M2**, oxygen consumption over time (LD–DD); **M3**, oxygen consumption over time (LD ½); **M4**, oxygen consumption over light-phase duration (LD–LD ½); **M5**, MDH activity over time (LD–DD).*

Following the raw data (empty circles in Figures 2A,B), at the beginning of the experiment (month 0, December) the average length of individual krill in LD was 34.84 ± 0.70 mm (mean ± SEM). Please note that means ± SEM are not represented in Figures 2A,B. This was done in order to avoid overloading the figure, where we preferred to represent the confidence intervals around the model fit instead (dotted lines). A summary of the raw data with calculated monthly mean values and SEM can be found in Supplementary Table S2. The initial mean length value in LD was in agreement with that in DD in the same month (month 0, December: 35.61 ± 0.90 mm). After that, in LD krill body length remained constant until month 3 (March, mean body length = 35.31 ± 0.33 mm), then decreased during months 4, 5, and 6 (April, May, June) and reached a minimum in month 7 (July, mean body length = 27.75 ± 1.02 mm). In DD, krill body length started decreasing already during month 1, 2, 3, and 4 (January, February, March, and April), reaching a minimum in month 5 (May, mean body length = 29.48 ± 1.04 mm). After the minimum was reached, in LD krill body length increased steeply during months 8, 9, 10, and 11 (August, September, October, and November), reaching maximum values in month 12 (December, mean body length = 47.61 ± 1.40 mm), whereas in DD the increase in body length started in month 6 (June) and proceeded at a slower pace until month 12 (December), reaching a final mean value of 44.49 ± 0.84 mm.

### Temporal Patterns of Oxygen Consumption

#### LD and DD Treatments

**FIGURE 3:**
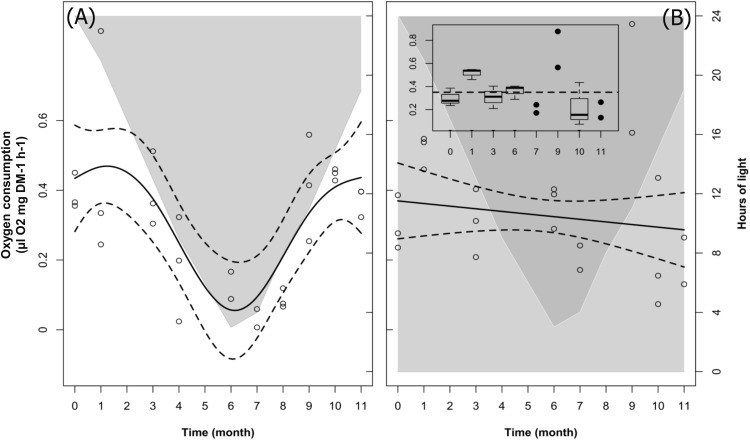
Changes in krill oxygen consumption over time in the LD (**A**) and DD (**B**) treatments. Solid dark lines represent the model fit; dotted lines represent confidence intervals around the fit; circles represent individual measurements. In LD **(A)**, light-gray shaded area vs. white area represents temporal changes in the duration of dark phase vs. light phase. In DD **(B)**, dark-gray shaded area vs. light-gray shaded area represents temporal changes in the duration of dark phase vs. subjective light phase. In DD **(B)**, upper-left small pane represents monthly changes in oxygen consumption relative to the treatment mean (dotted line). Please note that for months *n*°7, 9, and 11 we had only two observations (*n* = 2), and therefore we represented the raw values (as black dots) instead of the box and whiskers.

The changes over time in krill oxygen consumption were significant in LD but not in DD (Table 2, M2). This was confirmed also by the model fit comparison, which showed that the two model fits were significantly different from each other (Model fit comparison with Anova: *F*-value = 6.59; *p*-value = 0.014). Following the model fit (solid line in Figure 3A), the temporal pattern in LD was characterized by a decrease in oxygen consumption during the first half of the experiment followed by an increase during the second half. Following the raw data (empty circles in Figure 3A), oxygen uptake was maximal from month 0 (December, mean oxygen consumption ± SEM = 0.39 ± 0.02 μl O_2_^∗^mg^-1^ DM ^∗^ h^-1^) until month 3 (March, 0.39 ± 0.05 μl O_2_^∗^mg^-1^ DM ^∗^ h^-1^), then decreased and reached a minimum in month 7 (July, 0.03 ± 0.02 μl O_2_^∗^mg^-1^ DM ^∗^ h^-1^). After that, oxygen consumption remained low during month 8 (August, 0.09 ± 0.01 μl O_2_^∗^mg^-1^ DM ^∗^ h^-1^), then quickly recovered back to maximal levels already by month 9 (September, 0.41 ± 0.07 μl O_2_^∗^mg^-1^ DM ^∗^ h^-1^), remaining high until the end of the experiment (month 11, November, 0.37 ± 0.02 μl O_2_^∗^mg^-1^ DM ^∗^ h^-1^; month 12 is missing). Please note that as pointed out for Figures 2A,B in the section “Temporal Patterns of Krill Body Length in LD and DD,” also in Figures 3A,B we did not represent mean values ± SEM, but we reported the confidence intervals around the model fit instead (dotted lines). In addition, in Figure 3B we included a small pane representing a box-and-whiskers plot of the raw data in the different months. This was done in order to put in evidence the increase in oxygen consumption observed in month 1 (January) and month 9 (September) respect to the treatment average (dotted line in the small pane of Figure 3B). A summary of the raw data with calculated monthly mean values and SEM can be found in Supplementary Table S2.

In contrast to the temporal patterns observed in LD, no significant changes over time were detected in oxygen consumption in the DD treatment (Figure 3B, large pane), possibly due to great variability between months (Figure 3B, small pane). Following the raw data (empty circles in Figure 3B), at the beginning of the experiment (month 0, December), mean oxygen consumption ± SEM was lower compared to the same month in LD (0.30 ± 0.04 μl O_2_^∗^mg^-1^ DM ^∗^ h^-1^). In month 1 (January), we observed a sudden increase up to and above LD maximum levels (0.51 ± 0.02 μl O_2_^∗^mg^-1^ DM ^∗^ h^-1^). After that, oxygen consumption decreased to 0.31 ± 0.05 μl O_2_^∗^mg^-1^ DM ^∗^ h^-1^ in month 3 (March) and fluctuated back to 0.36 ± 0.03 μl O_2_^∗^mg^-1^ DM ^∗^ h^-1^ by month 6 (June). Values dropped again in month 7 (July, 0.21 ± 0.02 μl O_2_^∗^mg^-1^ DM ^∗^ h^-1^) and then increased suddenly in month 9 (September), again up to above LD maximum levels (0.72 ± 0.11 μl O_2_^∗^mg^-1^ DM ^∗^ h^-1^). After that, oxygen consumption dropped back to LD minimum-like values in month 11 (November, 0.22 ± 0.09 μl O_2_^∗^mg^-1^ DM ^∗^ h^-1^) and month 12 (December, 0.20 ± 0.05 μl O_2_^∗^mg^-1^ DM ^∗^ h^-1^).

#### LD1/2 Treatment

**FIGURE 4:**
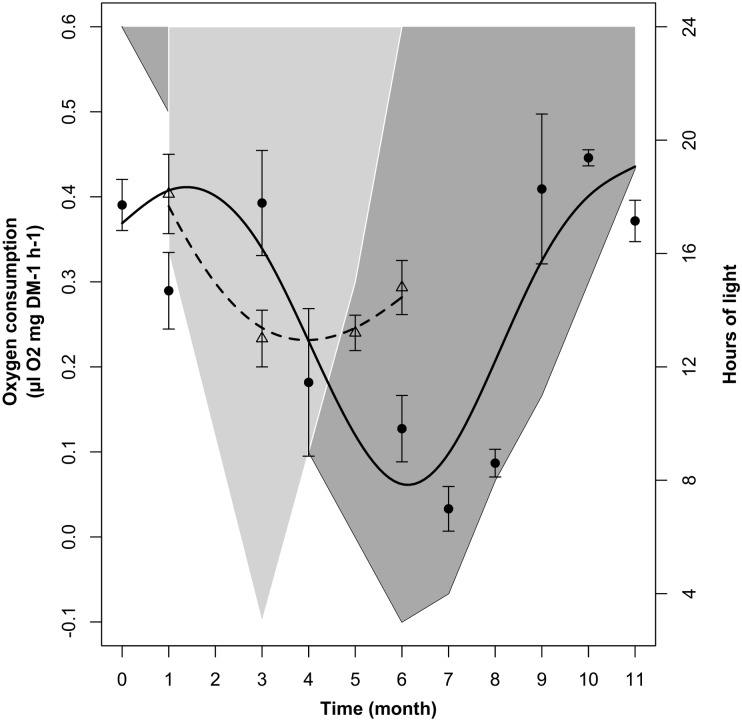
Changes in krill oxygen consumption over time in the LD1/2 treatment (dotted line represents the model fit; empty triangles represent monthly average ± SEM, *n* = 3) compared to the LD treatment (solid line represents the model fit, black dots represent monthly average ± SEM, *n* = 3). Light-gray shaded area represents changes in dark phase duration in LD1/2, dark-gray shaded area vs. white area represents dark phase vs. light phase duration in LD.

In the LD1/2 treatment there were significant changes in krill oxygen consumption over time (Table 2, M3). Following the model fit (dotted line in Figure 4), in LD1/2 oxygen consumption decreased during the first half of the experiment and slightly increased during the second half. Following the raw data, mean oxygen consumption ± SEM (empty triangles with associated error bars in Figure 4) was maximal in month 1 (January, 0.41 ± 0.04 μl O_2_^∗^mg^-1^ DM ^∗^ h^-1^; month 0 is missing), then dropped to minimum values in month 3 (March, 0.23 ± 0.03 μl O_2_^∗^mg^-1^ DM ^∗^ h^-1^). After that, oxygen consumption remained low in month 5 (May, 0.24 ± 0.02 μl O_2_^∗^mg^-1^ DM ^∗^ h^-1^) and finally showed a small increase in month 6 at the end of the experiment (June, 0.29 ± 0.03 μl O_2_^∗^mg^-1^ DM ^∗^ h^-1^). Please note that in Figure 4, unlikely Figures 2A,B, 3A,B, we did not represent the confidence intervals around the model fits, but we represented mean oxygen consumption values ± SEM instead. This was done to improve the readability of the figure, where the LD and LD1/2 treatments are over-imposed to favor an intuitive comparison between the two treatments. A summary of the raw data with calculated monthly mean values and SEM can be found in Supplementary Table S2.

The comparisons between LD and LD1/2 mean oxygen consumption levels in months 1 (January), 3 (March), and 6 (June) did not show significant differences, nevertheless the two treatments showed significant differences in the way oxygen consumption was influenced by light-phase duration in the different months (see section “Relationship Between Light-Phase Duration and Oxygen Consumption in LD and LD1/2”).

### Relationship Between Light-Phase Duration and Oxygen Consumption in LD and LD1/2

**FIGURE 5:**
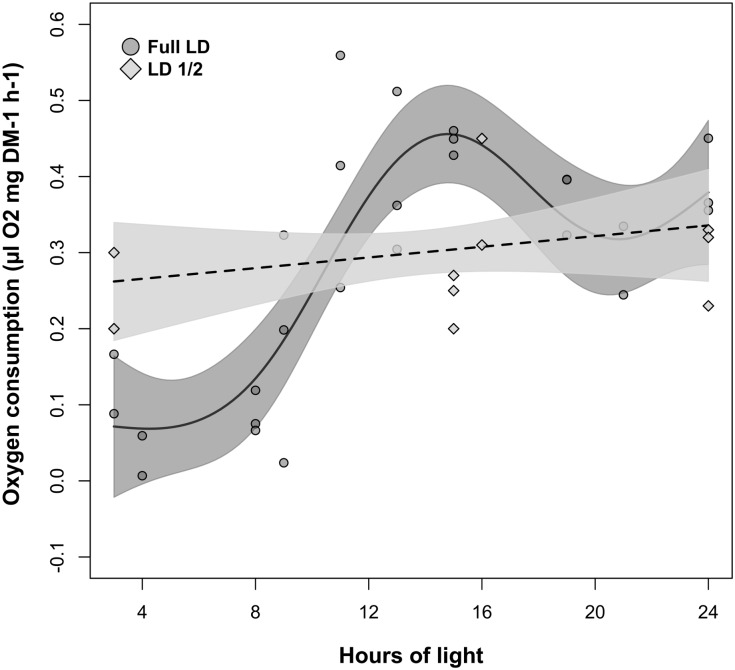
Changes in krill oxygen consumption over light-phase duration in the LD and LD1/2 treatments. For LD, solid line represents the model fit, dark-gray shaded area represents confidence interval around the fit, and dark-gray shaded circles represent individual measurements. For LD1/2, dotted line represents the model fit, light-gray shaded area represents confidence interval around the fit, and light-gray shaded squares represent individual measurements.

The relationship between oxygen consumption and light-phase duration was significant in LD but not in LD1/2 (Table 2, M4). This was confirmed also by the model fit comparison, which showed that the two model fits were significantly different from each other (Model fit comparison with Anova: *F*-value = 60.559; *p*-value = 0.001). In LD (Figure 5, solid line), oxygen consumption levels were constantly minimal when light-phase duration was between 3 and 8 h (light phase never went below 3 h). Between 8 and 16 h of light, oxygen consumption levels displayed a strong positive relationship with light-phase duration. Maximal oxygen consumption levels were observed when light-phase duration was about 16 h. Further increase in light-phase duration beyond 16 h did not result in a further increase in oxygen consumption, but in a small contraction instead. However, in the LD1/2 treatment no clear relationship between the two variables could be observed (Figure 5, dotted line).

### Temporal Patterns of MDH Activity in LD and DD

**FIGURE 6:**
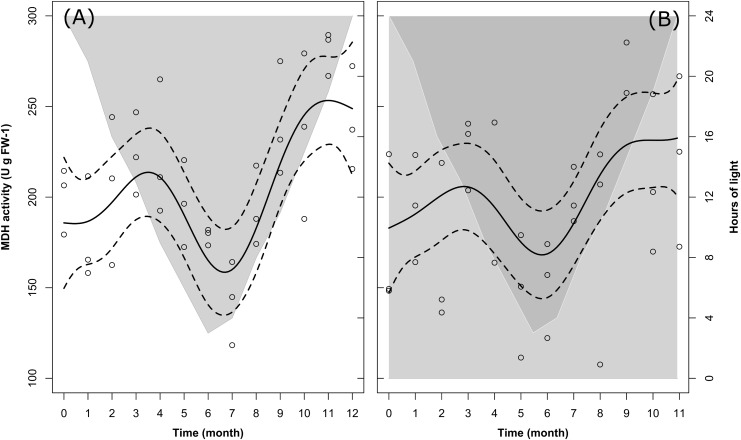
Changes in krill MDH activity over time in the LD **(A)** and DD **(B)** treatments. Solid dark lines represent the model fit; dotted lines represent confidence interval around the fit; circles represent individual measurements. In LD **(A)**, light-gray shaded area vs. white area represents temporal changes in the duration of dark phase vs. light phase. In DD **(B)**, dark-gray shaded area vs. light-gray shaded area represents temporal changes in the duration of dark phase vs. subjective light phase.

Changes in MDH activity over time were significant in LD and marginally non-significant in DD (Table 2, M5). Nevertheless, the model fit comparison showed that the two model fits were not significantly different from each other (Model fit comparison with Anova: *F*-value = 1.11; *p*-value = 0.36). In fact, following the model fit, in both treatments we observed a similar seasonal trend (solid lines in Figures 6A,B) characterized by at first, an increase in MDH activity between month 0 (December) and month 3 (March), then a drop between month 3 and month 6–7 (June–July), and then a second increase between month 6–7 and month 9–11 (September-November). The values then remained stable until the end of the experiment (month 12 in LD, month 11 in DD, 12 is missing).

Following the raw data (empty circles in Figures 6A,B), in LD mean MDH activity ± SEM at the beginning of the experiment (month 0, December) was 200.09 ± 8.68 U g FW^-1^, slightly higher than in DD at the same time of the year (173.79 ± 20.37 U g FW^-1^). In both treatments, the first maximum was reached in month 3 (March), with comparable mean values of 223.40 ± 10.72 U g FW^-1^ in LD and 226.37 ± 9.33 U g FW^-1^ in DD. After that, the winter minimum was reached in month 7 (July) in LD (142.44 ± 10.86 U g FW^-1^), whereas it was reached two months earlier (month 5, May) in DD (147.01 ± 15.99 U g FW^-1^). Similarly, the second maximum was reached in month 11 (November) in LD (281.05 ± 5.82 U g FW^-1^) and in month 9 (September) in DD (271.40 ± 9.84 U g FW^-1^). Please note that in Figures 6A,B, for the same reason as explained for Figures 2A,B, 3A,B, we did not represent mean values ± SEM, but we represented the confidence intervals around the model fit instead (dotted lines). A summary of the raw data with calculated monthly mean values and SEM can be found in Supplementary Table S2.

### Seasonal Changes in Gene Expression in LD and DD

#### Metabolic-Related Genes

**FIGURE 7:**
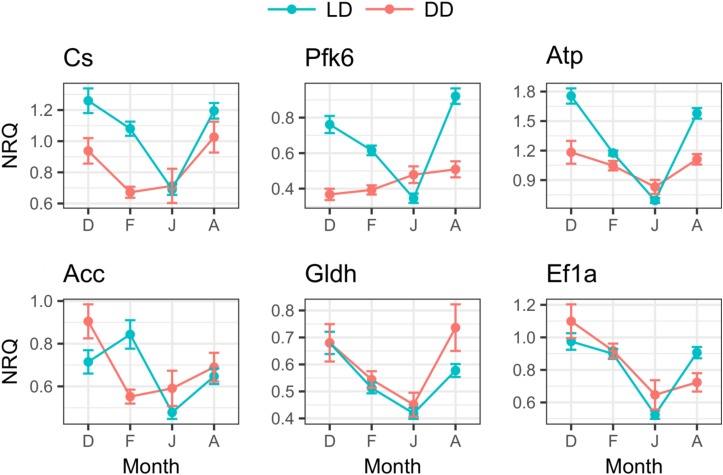
Seasonal changes of metabolic genes expression in LD and DD. For each month (D, December; F, February; J, June; A, August), mean NRQs ± SEM are reported. Sample sizes are as follows: December in DD, *n* = 16; December in LD, *n* = 47; February in DD, *n* = 48; February in LD, *n* = 48; June in DD, *n* = 16; June in LD, *n* = 43; August in DD, *n* = 16; August in LD, *n* = 48. For gene name abbreviations please see Supplementary Table S1.

In the LD treatment, all metabolic genes showed significant seasonal differences in their expression levels (Table 3), and all of them showed significant upregulation in December and August compared to June (*p* ≤ 0.005, Figure 7). Also, significant upregulation was generally observed in February compared to June (*p* ≤ 0.008, Figure 7). Moreover, *atp* and *gldh* showed significant upregulation in December compared to February (*p* ≤ 0.004, Figure 7), *pfk*6 showed significant upregulation in August compared to December (*p* ≤ 0.04, Figure 7), and *pfk*6 and *atp* also showed significant upregulation in August compared to February (*p* ≤ 0.001, Figure 7).

**Table 3:** Summary of results of Kruskal-Wallis (K-W) tests for differences in metabolic gene expression among seasonal samples in LD and DD.

	K-W	December–February	December–June	December–August	February–June	February–August	June–August
**LD**							
cs	<0.001	n.s.	<0.001	n.s.	<0.001	n.s.	<0.001
pfk6	<0.001	n.s.	<0.001	0.04	<0.001	<0.001	<0.001
atp	<0.001	<0.001	<0.001	n.s.	<0.001	<0.001	<0.001
acc	<0.001	n.s.	0.002	n.s.	<0.001	n.s.	0.005
gldh	<0.001	0.004	<0.001	n.s.	0.008	n.s.	<0.001
ef1a	<0.001	n.s.	<0.001	n.s.	<0.001	n.s.	<0.001
**DD**							
cs	0.009	0.02	n.s.	n.s.	n.s.	0.01	0.04
pfk6	n.s.	–	–	–	–	–	–
atp	0.03	n.s.	0.02	n.s.	n.s.	n.s.	0.02
acc	0.008	<0.001	0.01	n.s.	n.s.	n.s.	n.s.
gldh	0.02	n.s.	0.02	n.s.	n.s.	n.s.	0.02
ef1a	<0.001	n.s.	<0.001	0.02	<0.001	n.s.	n.s.


**P*-values were corrected using the false discovery rate (fdr) method. When K-W test was significant, *post hoc* pairwise comparisons between seasonal samples (December–February, December–June, December–August, February–June, February–August, June–August) were calculated after Dunn, and the *p*-values were corrected after Holm. Non-significant *p*-values (*p* ≥ 0.05) are indicated as n.s. For gene-name abbreviations please see Supplementary Table S1.*

In the DD treatment, five of the six tested genes showed significant seasonal differences (Table 3). *Cs* and *acc* showed significant upregulation in December compared to February (*p* ≤ 0.02, Figure 7); *atp*, *acc*, *gldh* and *ef*1a showed significant upregulation in December compared to June (*p* ≤ 0.02, Figure 7); *ef*1a showed upregulation in December compared to August (*p* ≤ 0.02, Figure 7), and also in February compared to June (*p* ≤ 0.001, Figure 7); *cs* showed upregulation in August compared to February (*p* ≤ 0.01, Figure 7); and *cs*, *atp*, and *gldh* showed upregulation in August compared to June (*p* ≤ 0.04, Figure 7).

Moreover, comparing the expression levels between LD and DD at each seasonal sampling time (December–February–June-August), we observed that *pfk*6 and *atp* showed significant upregulation in December in LD (Table 4 and Figure 7), *cs*, *pfk*6, *atp* and *acc* showed significant upregulation in February in LD (Table 4 and Figure 7), and *pfk*6 and *atp* showed significant upregulation in August in LD (Table 4 and Figure 7). No significant differences between LD and DD were observed in June (Table 4).

**Table 4:** Summary of results of Wilcoxon Rank Sum tests on differences in gene expression between the LD and DD treatment at the four seasonal sampling times (December, February, June, and August).

LD–DD	December	February	June	August
cs	n.s.	<0.001	n.s.	n.s.
pfk6	<0.001	<0.001	n.s.	<0.001
atp	0.001	0.01	n.s.	<0.001
acc	n.s.	0.004	n.s.	n.s.
gldh	n.s.	n.s.	n.s.	n.s.
ef1a	n.s.	n.s.	n.s.	n.s.
clk	n.s.	<0.001	n.s.	0.001
cry2	n.s.	<0.001	n.s.	n.s.
tim1	n.s.	<0.001	n.s.	n.s.
rh1a	0.02	<0.001	n.s.	n.s.
rh6	n.s.	n.s.	n.s.	n.s.
rrh	n.s.	<0.001	n.s.	n.s.


**P*-values were corrected after Holm. Non-significant *p*-values (*p* ≥ 0.05) are indicated as n.s. For gene-name abbreviations please see Supplementary Table S1.*

#### Clock and Light-Related Genes

**FIGURE 8:**
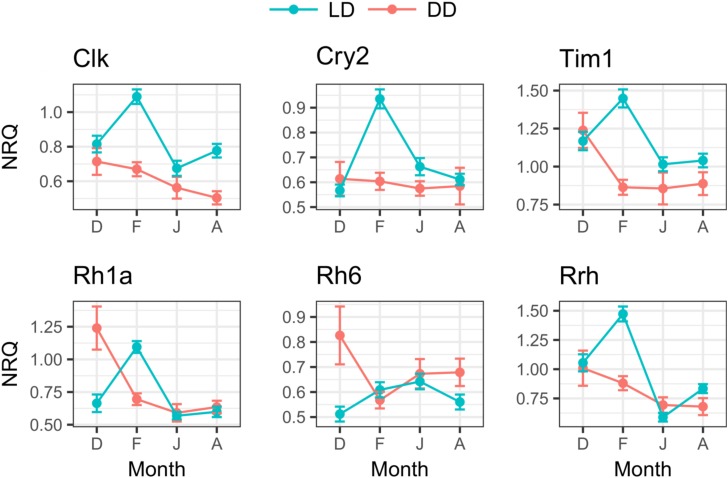
Seasonal changes of clock and light-related genes expression in LD and DD. For each month (D, December; F, February; J, June; A, August), mean NRQs ± SEM are reported. Sample sizes are as follows: December in DD, *n* = 16; December in LD, *n* = 47; February in DD, *n* = 48; February in LD, *n* = 48; June in DD, *n* = 16; June in LD, *n* = 43; August in DD, *n* = 16; August in LD, *n* = 48. For gene name abbreviations please see Supplementary Table S1.

In the LD treatment, all clock and light-related genes showed significant seasonal differences in their expression levels (Table 5). Upregulation was generally observed in February compared to December, June, and August (*p* ≤ 0.001, only *rh*6 did not show upregulation in February, Figure 8). *Rh*6 showed significant upregulation in June compared to December (*p* = 0.01, Figure 8), while *rrh* showed significant upregulation in December compared to June (*p* ≤ 0.001) and August (*p* = 0.03), and in August compared to June (*p* = 0.003, Figure 8).

**Table 5:** Summary of results of Kruskal-Wallis (K-W) tests for differences in clock and light-related gene expression among seasonal samples in LD and DD.

K-W	December–February	December–June	December–August	February–June	February–August	June–August
**LD**							
clk	<0.001	<0.001	n.s.	n.s.	<0.001	<0.001	n.s.
cry2	<0.001	<0.001	n.s.	n.s.	<0.001	<0.001	n.s.
tim1	<0.001	0.001	n.s.	n.s.	<0.001	<0.001	n.s.
rh1a	<0.001	<0.001	n.s.	n.s.	<0.001	<0.001	n.s.
rh6	0.01	n.s.	0.01	n.s.	n.s.	n.s.	n.s.
rrh	<0.001	<0.001	<0.001	0.03	<0.001	<0.001	0.003
**DD**				
clk	n.s.	–	–	–	–	–	–
cry2	n.s.	–	–	–	–	–	–
tim1	n.s.	–	–	–	–	–	–
rh1a	0.01	0.01	0.002	0.02	n.s.	n.s.	n.s.
rh6	n.s.	–	–	–	–	–	–
rrh	n.s.	–	–	–	–	–	–


**P*-values were corrected using the false discovery rate (fdr) method. When K-W test was significant, *post hoc* pairwise comparisons between seasonal samples (December–February, December–June, December–August, February–June, February–August, June–August) were calculated after Dunn, and the *p*-values were corrected after Holm. Non-significant *p*-values (*p* ≥ 0.05) are indicated as n.s. For gene-name abbreviations please see Supplementary Table S1.*

In DD, only *rh*1a showed significant seasonal differences (Table 5), with significant upregulation in December compared to February (*p* = 0.01), June (*p* = 0.002) and August (*p* = 0.02, Figure 8).

Comparing the expression levels between LD and DD at each seasonal sampling time (December–Feburary–June–August), we observed general upregulation in February in LD (Table 4, only *rh*6 did not show significant differences, Figure 8). Moreover, *rh*1a showed significant upregulation in DD in December (Table 4 and Figure 8), while *clk* showed upregulation in August in LD (Table 4 and Figure 8). No significant differences were observed between LD and DD in June (Table 4).

## Discussion

### Influence of Photoperiod and Food Availability on the Regulation of Krill Seasonal Metabolic Cycle

During our LD simulation, krill displayed clear seasonal cycles of growth, enzyme activity, oxygen consumption, and metabolic gene expression, with a trough in June–July at simulated 66°S midwinter light conditions and a peak in December–January at simulated 66°S midsummer light conditions. This was in agreement with field observations showing that krill undergo a period of winter dormancy to save energy during the food-depleted season, while they enhance their activity during summer to take advantage of the abundant food resources and trigger reproduction (Stepnik, 1982; Quetin and Ross, 1991; Meyer, 2012). Early observations by Ikeda and Dixon (1982) showing that laboratory krill starved for over 200 days did not die but responded with body shrinkage instead, suggested that major physiological changes related to krill winter dormancy might be directly regulated by food availability. However, Kawaguchi et al. (1986) and Torres et al. (1994) later observed that seasonal changes in krill metabolic activity did not always correlate with changes in food availability, suggesting a different mechanism of regulation. Indeed, following laboratory experimentations showed that krill response to high food conditions during winter could be influenced by changes in light regime, and that prolonged photoperiods could stimulate increased feeding activity (Atkinson et al., 2002; Teschke et al., 2007; Meyer et al., 2010). This suggested that krill metabolic cycle was not directly regulated by seasonal changes in food availability, but rather by changes in feeding activity stimulated by the Antarctic light regime (Meyer et al., 2010). The results obtained during our LD simulation under constant food conditions indicated that food availability was not the main factor influencing krill metabolic cycle and supported the hypothesis that the seasonal light-regime was playing a major role instead.

### Endogenous Regulation of Krill Metabolic Cycle

Based on the observation that different light regimes could affect the feeding activity of krill during winter (Teschke et al., 2007), Meyer et al. (2010) hypothesized the presence in krill of an inherent (i.e., endogenous) seasonal metabolic cycle triggered by photoperiodic cues. This hypothesis was further investigated by Brown et al. (2013), who showed that krill oxygen consumption increased after winter, independently from light regime, food availability and temperature, suggesting the presence of an endogenous seasonal rhythm which had been entrained prior the commencement of the experiment. Indeed, the seasonal cycles of growth, enzyme activity and gene expression observed during our DD simulation strongly supported this hypothesis, indicating that fundamental processes related to krill growth and metabolism were following an inherent seasonal cycle irrespective of light and food conditions.

However, krill oxygen consumption, which is generally used as a proxy for overall metabolic activity, showed a seasonal cycle only in LD. Oxygen consumption represents the sum of multiple physiological processes occurring together, including basal metabolism, swimming activity, growth, feeding, and reproduction (Clarke and Morris, 1983). Therefore, the correlation between the seasonal cycles of oxygen consumption, growth, and enzyme activity that we observed in LD was in some ways to be expected. Conversely, the missing correlation of oxygen consumption observed in DD would suggest that the seasonal dynamics of some oxygen-demanding process was altered by the prolonged exposure to constant darkness. Considering the endogenous seasonal patterns displayed in DD by krill growth and by the expression profile of *ef*1a, a gene involved in protein synthesis and cell growth and proliferation, we might suggest that growth-related processes were not directly affected. Similarly, considering the endogenous cycles displayed in DD by MDH activity and by the expression profiles of *cs* (involved in the Citric Acid Cycle) and *atp* (involved in ATP synthesis), we might suggest that basal carbohydrate and energy metabolism were also not directly affected. However, this would be opposed by the different response displayed in LD and DD by the metabolic gene *pfk*6, coding for the rate-limiting step of the glycolytic pathway, which showed a clear seasonal profile only in LD. In this case, we might hypothesize that basal krill metabolic functions related to glycolysis were indeed being altered under prolonged darkness, possibly leading to the different oxygen consumption pattern observed in DD. In fact, even if glycolysis is generally considered as an oxygen-independent metabolic pathway, it may still contribute to overall aerobic metabolism since its end products (pyruvate and reducing power) can be used to fuel the Citric Acid Cycle, which is a typical oxygen-dependent process.

### Reaction to a Shift in the Seasonal Photoperiodic Cycle

Even if no overt seasonal cycle of oxygen consumption was observed in DD, the comparison between the oxygen consumption pattern observed in LD and that observed in LD1/2 suggested the interplay of a seasonal (i.e., endogenous) factor in the regulatory process. In fact, despite that clear temporal changes in oxygen consumption were observed in both treatments, significant differences were present in the relationship between light-phase duration and oxygen consumption between the two treatments. In particular, this seemed to be related to the different rate of change of oxygen consumption observed in LD1/2, which did not compensate for the rate of advance in the photoperiodic cycle. This was especially evident in May and June, when oxygen consumption was still at minimal levels despite the fact that a significant increase in light-phase duration had already taken place (from 3 h in March to 15 h in May and 24 h in June). In this sense, the results suggested that the seasonal metabolic cycle in krill is regulated by an underlying endogenous factor which is only slightly affected by a major shift in the photoperiodic cycle.

### Photoperiodic Entrainment of Krill Seasonal Metabolic Cycle

Following the hypothesis that krill seasonal metabolic cycle was regulated by an endogenous timekeeping mechanism (i.e., a circannual clock) entrained by light regime (Meyer et al., 2010; Brown et al., 2013), the lack of seasonality observed for oxygen consumption in DD might be interpreted as a lack of proper photoperiodic entrainment. In fact, in order to achieve effective circannual synchronization, the onset of a seasonal response might require exposition to specific photoperiodic cues during a determined time of the year, which in the case of a winter-related response may be represented by the gradual shortening of day length at the end of summer (Woodfill et al., 1994). Since our DD treatment was initiated early in December (midsummer), this would have deprived krill of those late summer/early autumn light cues which might have been necessary to entrain their circannual clock and elicit proper orchestration of the winter-related responses, possibly causing an uncoupling among the different oxygen-demanding processes. This would be in agreement with the different seasonal response observed by Brown et al. (2013), who initiated their DD treatment later in autumn thus allowing for proper krill entrainment during the previous summer.

The presence of specific photoperiodic thresholds (or conditions) for the timing of krill seasonal metabolic responses was suggested also by the light-oxygen relationship observed in the LD treatment. In fact, metabolic down-regulation toward winter was initiated only after the light phase duration had been reduced below 16 h, corresponding to an early autumn light regime, whereas metabolic up-regulation occurred only after light phase duration had exceeded 8 h, corresponding to a late winter light regime. A similar season-specific interaction with light regime was also displayed by metabolic gene expression. In fact, while the DD expression levels of *pfk*6, *cs* and *acc* in February were already minimal and comparable with June (i.e., winter-like), those in LD were still maximal and comparable with December (i.e., summer-like), suggesting that early autumn light conditions might represent a threshold also at the transcriptional level. Conversely, an anticipatory (e.g., endogenous) mechanism seemed to take place at the molecular level at the end of winter, as metabolic gene expression increased to summer-like values already by August, when oxygen consumption was still minimal. This suggested that while the initiation of krill winter dormancy might be regulated in a top-down manner, with the environmental changes (i.e., light regime) triggering the physiological responses, the termination of krill winter dormancy might be regulated in a bottom-up manner, with the physiological responses being triggered by the endogenous factors. A similar concept was suggested also by Brown et al. (2011) regarding the regulation of krill seasonal sexual maturity cycle, and might therefore represent a general mechanism underlying krill seasonal timekeeping.

### Involvement of Circadian Clock Genes

Photoperiodic time-measurement can be achieved through the interaction between appropriate light cues and an internal timekeeping mechanism (i.e., a photoperiodic clock), which can track changes in day length over a period of time and stimulate the season-specific response (Dolezel, 2015). Even if the nature of the photoperiodic clock remains elusive, various evidences have been collected indicating the involvement of elements of the circadian clock. The circadian clock is a molecular oscillator based on rhythmic clock gene expression, which can be entrained by light cues and can stimulate daily rhythms in metabolism and behavior (Dunlap, 1999). Being able to track day length, the circadian clock has the potential to play a role in photoperiodic time-measurement, and the link between clock gene expression and the regulation of seasonal responses has indeed been demonstrated in mammals and insects (Hazlerigg, 2010; Goto, 2013). In krill, the circadian clock has been involved in the regulation of daily rhythms in behavior, metabolism and transcription (Gaten et al., 2008; Teschke et al., 2011; De Pittá et al., 2013), but a putative clock involvement in the regulation of krill seasonal strategies has not yet been investigated.

During our simulation, the krill clock genes *clk*, *cry*2, and *tim*1 showed upregulation in autumn in LD, suggesting a specific link between clock-related activity and light regime at that time of the year. Interestingly, photoperiodic-dependent regulation of *tim*, *cry*2, and *clk* was found to play a major role in the induction of seasonal responses in the Drosophilid fly *Chymomyza costata* (Stehlík et al., 2008) and in the bean bug *Riptortus pedestris* (Ikeno et al., 2011, 2013), suggesting that a similar process may also take place in krill.

Since the light input to the photoperiodic clock might be transmitted by elements of the phototransduction cascade (Tamaki et al., 2013), we also monitored seasonal changes in the expression levels of the krill opsins *rh*1a, *rh*6, and *rrh*, which had previously shown rhythmic expression at the daily level (De Pittá et al., 2013; Biscontin et al., 2016). Interestingly, *rh*1a and *rrh* showed LD autumn upregulation in a similar way to the clock genes, suggesting the presence of a signaling cascade linking the photoperiodic cue to the putative molecular timing mechanism.

### Ecological Implications for Krill in the Field

Due to the marked seasonal photoperiodic cycle at high-latitudes, regulation of seasonal phenology via photoperiodic time-measurement would probably represent the most intuitive timekeeping system for krill, allowing for reliable synchronization of major life-cycle events with the seasonal time course in the environment (Gwinner, 2012). However, the seasonal photoperiodic signal might vary significantly across the broad latitudinal range of krill distribution (approx. from 51 to 70°S), also considering that individual krill might be capable of actively and/or passively traveling across many degrees of latitude and move among different habitats (open ocean, continental shelf, sea-ice marginal zone, deep ocean sea floor, etc.) within and between seasons (Thorpe et al., 2007; Reiss et al., 2017). The influence of photoperiod might become less stringent when krill move toward lower latitudes, or alternatively the photoperiodic threshold (if present) might show a latitudinal cline, for example getting longer (in terms of hours of light) at higher latitudes (Helm et al., 2013). The effect of such latitudinal shifts on the photoperiodic regulation of krill seasonal responses is still unclear, but Höring et al. (2018) recently found that krill maturity cycle showed differences in critical photoperiod (i.e., the photoperiod when 50% of individual krill were fully mature) according to different simulated latitudinal light regimes, suggesting the presence in krill of a flexible endogenous timing system.

This might represent an important adaptation, allowing krill to adjust their seasonal responses depending on the local conditions, as suggested before for the organization of seasonal responses in birds (Visser et al., 2010; Helm et al., 2013). With respect to this, Schmidt et al. (2014) showed that winter food uptake (used as an indicator of overall metabolic activity) differed significantly between krill collected at similar latitudes (60–65°S) but in different habitats (the Lazarev Sea, an ice-covered and food-depleted deep oceanic area, and the Bransfield Strait, an ice-free and highly productive shelf area), suggesting the presence of specific regulatory cues operating at the local scale. Conversely, Seear et al. (2012) found no differences between the expression levels of genes involved in respiration and motor activity in krill collected during winter at the Antarctic Peninsula (62°S) and South Georgia (54°S), suggesting that both populations were in a similar state of quiescence despite the different latitude. However, in the South Georgia region, where more favorable growth and feeding conditions were present, krill showed up-regulation of genes involved in feeding, digestion, and immunity, suggesting enhanced levels of feeding activity (Seear et al., 2012). A hierarchy of external signals might therefore interact with photoperiod to modulate the seasonal life-cycle of krill at the local scale, and food availability might be the most influent among them. To understand if food availability may regulate the seasonal shifts in krill metabolic activity in a similar way as observed here for photoperiod, new experiments should be conducted with krill exposed to constant photoperiods and seasonally changing food conditions.

Finally, two of the major krill feeding grounds in the Southern Ocean, the southwest Atlantic sector and the region of the West Antarctic Peninsula, are currently experiencing rapid anthropogenic-driven warming (Meredith and King, 2005), and the abundance of krill in these areas has already been altered significantly as a result of changes in primary productivity associated with sea-ice decline (Atkinson et al., 2004). In this context, the implications for krill to entrain their seasonal responses through photoperiod might become problematic, as krill photoperiodic-driven metabolic cycle might have evolved to anticipate seasonal changes in phytoplankton blooms and optimize the match with food sources at the end of winter. Phytoplankton dynamics might be affected by climate change, delineating a “match–mismatch” scenario in which krill seasonal timing might need to be rescheduled, with potential negative effects on the krill population and on the rest of the ecosystem.

## Conclusion

Our results supported the hypothesis that krill seasonal metabolic cycle was not regulated by changes in food availability, but rather by an endogenous timing system entrained by photoperiod instead. Endogenous regulation was observed in relation to krill growth, enzyme activity and gene expression. However, photoperiodic entrainment seemed to play a major role in the regulation of krill oxygen consumption. We hypothesize that specific light cues presented to krill during autumn might be necessary for the entrainment of the seasonal metabolic cycle. The light input might be collected by specific elements of krill phototransduction cascade and transmitted to an internal photoperiodic clock. The nature of such clock still remains elusive, but we suggest that elements of krill circadian clock might be involved.

## Author Contributions

MT, BM, and SK conceived and designed the study. MT and RK contributed to the maintenance of the long-term simulations, the collection of sample and the measurements of krill body length. MT measured krill oxygen consumption and MDH activity. FP measured krill gene expression. FP and LS performed the statistical analysis. MT and FP wrote the first draft of the manuscript. All authors contributed to manuscript revision, read and approved the submitted version.

## Conflict of Interest Statement

The authors declare that the research was conducted in the absence of any commercial or financial relationships that could be construed as a potential conflict of interest.
