# Supplementary material for: The Seasonal Metabolic Activity Cycle of Antarctic Krill (Euphausia superba): Evidence for a Role of Photoperiod in the Regulation of Endogenous Rhythmicity
Source: Front Physiol. 2018 Dec 20;9:1715. doi: 10.3389/fphys.2018.01715 (PMC6307472; doi:10.3389/fphys.2018.01715)
Supplement: Supplementary file 1 [file Table_1.PDF]

Supplementary Table 1

Summary information of selected target metabolic, clock and light-related genes, and candidate reference genes (hk)

	Gene Name	Short Form	Forward Primer Sequence 5'-3'	Reverse Primer Sequence 5'-3'	Internal Probe Sequence 5'-3'	Sequence ID
**metabolism**	citrate synthase	cs	ATGTCTCGCTGCTGCTATCTC	TCTTCACTAGTTATTGTTGATGCCAGAA	CAAGTCCTCAAAATTC	ESS076337
	ATP synthase subunit gamma	atp	GTCAAGAACATCCAGAAGATCACTCA	GCTTCAACTCCCTTTCAGCTCTT	CTGCTGCCAAGTTTGC	ESS129614
	phosphofructokinase-6	pfk6	AGTTATAACAAGAGTTAAGCAGCAGTGT	CCCTGACATGCCAAAAATGCA	ATACCGACCAAAATCA	ESS011313
	acetyl-CoA carboxylase	acc	TGCAGACGCCTCAAGTTGA	GTTTGTTAGCGCCACAGTTTGT	AACAACGCCAGACCTT	ESS011644
	glutamate dehydrogenase	gldh	CTGTTCAATCCTTGTCAAGATGCTT	TGTACTGGCACTGGCTTATATTGC	CCAAGCAACAATACCC	ESS028087
	elongation factor 1-alpha	ef1a	TGGGCAAGGAAAAGATCCACATC	CTTGCCGGAGTCGACATGA	CCAACCACCACAATCG	ESS116888
**clock**	clock	clk	GGCCTCAGTTGGTACGAGAAATG	AATTTCCATTCTATACTGTGCCTTGATGT	TTGGCTCCAGAATCAT	ESS034513
	cryptochrome 2	cry2	CAGTGCTCAAGAACTTCCCAACTAA	GCGTCCTATGACACATTTAGACTGT	ACTGCACCAGAAAAT	ESS118469
	timeless 1	tim1	CAAGACAAAGCGAGATGGCATTT	AGGGTTGGAAGAAGGTTTTGTGAAA	TCGGCGTTCACTCTTC	ESS040526
**light**	medium-wavelength sensitive opsin	rh1a	TCATTGATAAGCATTGGGCCAACT	GCCAAGGACATAGTGCCACAT	TCCCACCTGTGAACCC	ESS058227
	long-wavelength sensitive opsin	rh6	GTGTATGCCATTAGTCATCCCAAGT	ACTGAGCCATGGCATTTTCGT	CCGTGCTGCCCTCTAT	ESS092680
	peropsin	rrh	CTGCTGTTGGTGCCATGATC	TGTGATGCACTACTTTCTCTTAGATTCAG	CTCCACAGGAACTTCT	ESS008171
**hk**	ribosomal protein S13	rpS13	TGCTCAGGTTCGCTTTGTCA	CTCTGGGATATCTGGAGCAAGTC	CACACGCAGGATTTT	ESS060818
	ribosomal protein L32	rpL32	TCAAGCCTAACTGGCGTAAGC	TGACCCTTGAAGCGACGAC	CCTGTTGTCAATACCC	ESS059136
	ubiquitin carboxyl-terminal hydrolase 46	usp46	TGGAACTGGTATTAACAGAGGACACT	ACTGCATCGTCATCAAAGAGCA	AAGAGCCACAGATTTT	ESS079224

all sequences available online at http://krilldb.bio.unipd.it/
