# Supplementary material for: The Seasonal Metabolic Activity Cycle of Antarctic Krill (Euphausia superba): Evidence for a Role of Photoperiod in the Regulation of Endogenous Rhythmicity
Source: Front Physiol. 2018 Dec 20;9:1715. doi: 10.3389/fphys.2018.01715 (PMC6307472; doi:10.3389/fphys.2018.01715)
Supplement: Supplementary file 2 [file Data_Sheet_1.PDF]

Supplementary Table 2

Summary of raw data regarding body length, oxygen consumption and MDH activity measurements in the three treatments (LD, DD and LD1/2)

		body length (mm)			oxygen consumption (µl O2 *mg-1 DM * h-1)			MDH activity (U g fw-1)		
treatment	month	individual measurements	monthly mean	SEM	individual measurements	monthly mean	SEM	individual measurements	monthly mean	SEM
LD	0	33.33	34.84	0.70	0.45	0.39	0.02	179.34	200.09	8.68
		34.87			0.37			214.47		
		36.32			0.36			206.47		
	1	33.99	33.45	0.49	0.86	0.48	0.16	158.13	178.37	13.65
		32.24			0.24			165.47		
		34.12			0.33			211.53		
	2	40.44	34.70	2.58	NA	NA	NA	210.32	205.67	19.33
		29.54			NA			162.53		
		34.11			NA			244.16		
	3	36.07	35.31	0.33	0.36	0.39	0.05	201.40	223.40	10.72
		35.16			0.30			246.81		
		34.71			0.51			221.99		
	4	34.08	32.97	0.77	0.20	0.18	0.07	264.97	222.83	17.75
		33.74			0.02			210.98		
		31.09			0.32			192.52		
	5	36.60	33.26	1.77	NA	NA	NA	220.41	196.40	11.31
		33.98			NA			196.34		
		29.21			NA			172.44		
	6	32.59	32.48	0.68	0.17	0.13	0.03	181.91	178.51	2.13
		33.86			NA			173.40		
		30.99			0.09			180.22		
	7	29.93	27.75	1.02	NA	0.03	0.02	144.84	142.44	10.86
		27.71			0.06			164.18		
		25.60			0.01			118.30		
	8	38.03	38.01	0.28	0.08	0.09	0.01	188.00	193.18	10.40
		38.59			0.12			174.17		
		37.40			0.07			217.37		
	9	38.05	38.06	0.88	0.56	0.41	0.07	231.76	240.06	14.90
		36.20			0.25			275.00		
		39.94			0.41			213.44		
	10	41.42	41.78	0.75	0.46	0.45	0.01	187.97	235.37	21.57
		43.52			0.45			238.84		
		40.41			0.43			279.29		
	11	47.57	46.10	1.18	0.40	0.37	0.02	266.88	281.05	5.82
		47.51			0.32			286.81		
		43.22			0.40			289.46		
	12	44.18	47.61	1.40	NA	NA	NA	215.53	241.68	13.50
		49.42			NA			237.22		
		49.24			NA			272.28		
DD	0	37.58	35.61	0.90	0.24	0.30	0.04	149.35	173.79	20.37
		33.78			0.39			223.68		
		35.46			0.28			148.33		
	1	35.40	34.89	0.80	0.46	0.51	0.02	195.35	194.23	13.95
		36.27			0.55			223.24		
		33.00			0.54			164.11		
	2	33.39	34.46	0.52	NA	NA	NA	136.32	166.23	21.56
		34.38			NA			218.87		
		35.60			NA			143.48		
	3	33.58	34.18	0.35	0.40	0.31	0.05	234.88	226.37	9.33
		35.02			0.31			240.49		
		33.95			0.21			203.75		
	4	NA	32.80	0.59	NA	NA	NA	NA	202.48	27.34
		33.63			NA			241.15		
		31.96			NA			163.82		
	5	30.35	29.48	1.04	NA	NA	NA	179.01	147.00	15.99
		26.98			NA			111.44		
		31.11			NA			150.56		
	6	36.17	34.23	1.47	0.29	0.36	0.03	122.23	151.10	12.45
		30.64			0.39			157.02		

		35.87			0.40			174.06		
	7	32.82	33.41	0.65	NA	0.21	0.02	216.71	199.59	7.28
		35.00			0.17			195.34		
		32.42			0.24			186.72		
	8	38.78	34.33	3.41	NA	NA	NA	223.57	179.39	29.56
		25.97			NA			107.62		
		38.23			NA			206.98		
	9	38.41	32.90	3.65	0.56	0.72	0.11	285.31	271.40	9.84
		24.06						NA		
		36.24			0.88			257.49		
	10	35.97	37.64	1.82	0.16	0.22	0.09	169.91	209.84	20.68
		34.88			0.07			256.79		
		42.06			0.44			202.83		
	11	41.42	41.22	0.91	0.13	0.20	0.05	266.70	221.45	22.22
		39.19			NA			225.04		
		43.04			0.26			172.62		
	12	44.21	44.49	0.84	NA	NA	NA	NA	NA	NA
		46.39			NA			NA		
		42.87			NA			NA		
LD1/2	0	NA			NA	NA	NA	NA		
		NA			NA			NA		
		NA			NA			NA		
	1	NA			0.45	0.41	0.04	NA		
		NA			0.45			NA		
		NA			0.31			NA		
	2	NA			NA	NA	NA	NA		
		NA			NA			NA		
		NA			NA			NA		
	3	NA			0.20	0.23	0.03	NA		
		NA			0.20			NA		
		NA			0.30			NA		
	4	NA			NA	NA	NA	NA		
		NA			NA			NA		
		NA			NA			NA		
	5	NA			0.25	0.24	0.02	NA		
		NA			0.20			NA		
		NA			0.27			NA		
	6	NA			0.33	0.29	0.03	NA		
		NA			0.32			NA		
		NA			0.23			NA		
